# Monthly Summary

**Published:** 1871-04

**Authors:** 


### MONTHLY SUMMARY.

*Bibliographical and Microscopical Section, Academy of Natural Sciences.*—At a meeting of the Section, held Monday, February, 6, the Director, S. W. Mitchell, M. D., in the chair.

Mr. Wm. H. Walmsley exhibited slides showing the difference between the torn edge of our ordinary paper and that of the thick, strong paper used by the Chinese for the manufacture of clothing. Both appeared to be of cotton, but the foreign article bore the aspect of being made directly from the cotton fibres instead of from comminuted woven fabrics.

Dr. J. H. McQuillen directed attention to the remarkable specimen of hypertrophy of the roots of a left superior first molar tooth measuring 2½ inches in length by 2⅝ inches in circumference, and weighing 12½ pennyweights, which he had exhibited at a previous meeting, and of which he had promised to make a microscopic examination. This he had done, and now submitted the results under the microscope, in three sections, taken from the same locality, one under the other.

As he had anticipated, the growth proved to be hypertrophy of the cementum.

The term exostosis, frequently applied to this growth, was open to exception on account of the fact that there are certain characteristic differences between cementum and bone, which any one at all familiar with the microscopical anatomy of these two structures will readily recognize.

With the view of making this difference apparent to those unacquainted with the subject, he had placed under one of the microscopes a section of bone. This specimen shows a transverse section of the Haversian canals, with the lacunæ and canaliculi arranged around them in concentric lamina. In the cementum, on the contrary, there is an entire absence of the Haversian canals, while the lacunæ and canaliculi are quite numerous.

The three sections from the hypertrophied structure differed from each other in a somewhat remarkable manner, when we take into consideration the fact that they had been in such close contiguity. In the first, an outer section, the lacunæ and canaliculi are presented in the usual arrangement of cementum. In the second section, taken immediately adjacent and below it, there are, in addition to the lacunæ and canaliculi, a number of irregular spaces; while in the third section the lacunæ are largely increased, and the canaliculi are more numerous and increased in length, running in a spiral direction analogous to dentinal tubuli in secondary dentine.

Dr. F. W. Lewis inquired what difference there was between these exostoses and epulis.

Dr. James Tyson replied that, microscopically, true epulis is a fibrous tissue, but that the term is often erroneously applied to the so-called myeloid tumors of Paget (the giant-celled sarcoma of Virchow) which have a similar situation,—that is, any of the structures about the jaw-bone, periosteum, and fibrous mucous membrane,—and which are cellular, or contain at most a small amount of fibrous tissue. Dr. T. also suggested that it seemed at least more natural to place cementum and bone in the same category, since the former differed from true osseous tissue only in the absence of the Haversian canals. the object of which was merely to contain the minute blood-vessels, which were here unnecessary on account of the close contiguity of the structure (cementum) to its vascular supply.

Dr. McQuillen stated that the various characteristics of enamel, cementum, and bone had induced Prof. Owen and others to draw the distinction which he had mentioned.

Dr. Tyson showed an ingenious gastric canula, improved by being fitted with a cover and catch, for preventing the loss of gastric juice by the animal scratching it open.—*Med. Times.*

---

*Function of the Prostate Gland.*—Dr. Kraus, editor of the Vienna *Medical Times*, contributed to the *Medical Times and Gazette*, February 11, 1871, a preliminary communication in regard to the seminal fluid and the influence of the prostatic secretion on its physical qualities. The conclusions arrived at are—1. The seminal fluid, as long as it remains within the testes, vesicles, and other seminal passages, is colorless and scentless, being in appearance exactly like fresh honey while deposited in the comb; and in its reactions it is neutral. 2. Only when it has quitted the passages and arrived in the urethra does it acquire its white color and its peculiar faint smell. 3. During its passage through the prostatic portion of the urethra, the prostate empties out its fluid, colors the semen white, and confers upon it the faculty of coagulating when exposed to the air (alkaline reaction). Semen taken from the seminal vesicles does not coagulate, but remains clear, colorless, and scentless. The spermatozoids, in the absence of the prostatic fluid, cannot live in the mucous membrane of the uterus of mammalia; but with its aid they may live for a long time in the uterine mucus, often more than thirty-six hours. From his experiments, which are not yet published, he draws the conclusion that the prostatic fluid exercises an unlimited influence on the viability of the spermatozoids, sustaining it when endangered by the mucus secreted by the mucous membrane of the uterus.

---

*Fibro-Serous Cyst of Jaw.*—Dr. Sayre exhibited, at a meeting of the N. Y. Pathological Society, a fibro-serous cyst removed from the inner angle of the left jaw of a gentleman aged 35. The tumor had been six years in growth, but had grown very slowly until last year; since then it has grown rapidly. (Two photographs of the case were exhibited, and the patient himself was also present.) Dr. Hamilton had seen the case in consultation, and had advised incision, on account of the depth of the tumor and the adhesions. Dr. Sayre decided, however, to perform excision, if possible. The adhesions were found more firm than had been anticipated, and there was some difficulty in dissecting the sac out. The jugular vein was exposed for $1\frac{3}{4}$ inch, but not injured. The fluid oozed out of the sac, during the operation, but it was again rendered tense by a silk ligature passed round it, and at last came out entire. No vessels were wounded, so no ligatures were required. The operation was performed last Sunday week. The incision was made parallel to the fibres of the sterno-cleido-mastoid muscle. Excision in this case proved better than incision.—*Medical Record.*

---

*Neuralgia of Jaw.*—The case to which I would first direct your attention this morning, is one of neuralgia of the jawbone, first specifically described by Professor Gross, in a paper read before the Surgical Section of the American Medical Association, at Washington, in May last. He attributes the disease to compression of the branches of nerves distributed through the wasted alveolar process depending on the enoachment of osseous matter upon the walls of the canals in which they are inclosed, in edentulous persons. It is most generally met with in elderly persons. It is more frequently seated in the upper than in the lower jaw. The morbid action is generally limited to the osseous structure, but not always. It can only be relieved by the removal of the affected alveolar process.

J. D——, a farmer, aged 63 years, has been suffering, for a number of years, with neuralgia of a most aggravating character, in the left side of the face. A year ago, I performed Carnochan's operation, of exsecting the second branch ot the fifth nerve of the left side with great relief. You see, now, the marks of that operation on his cheek.

Within a month, however, the neuralgia returned, and located itself in the left alveolar process of the upper jaw, and since then he has suffered the most excruciating paroxysms of pain, which last a few minutes, and then cease, to return again after a short interval. The paroxysms are always rendered worse by movements of the mouth, as in talking or eating. His general health is not good, his sleep is disturbed, his appetite is bad. Medicines have had no effect in even relieving his sufferings, and he has come to us again for surgical relief.

In examining his mouth, you will observe that all the teeth on the left side have been lost, and that the alveolar process, from the lateral incisor to the second molar, is enlarged, tuberculated, and as hard, apparently, as ivory. The remedy I will use, in this case, is excision of the affected alveolar process. I will make an incision along its ridge, from the incisor to the second molar tooth, turn aside the soft parts, and cut off the affected bone with bone forceps and gouge.

(The operation was performed as described. The bone removed was hard and eburnated. There was but little hemorrhage, and very little suffering afterwards. On the third day the patient left the hospital, having slept two nights without interruption.—*Surgical Clinic of* PROF. BRIGGS.—*Nashville Journal of Medicine and Surgery.*

---

*The Virtue of the Sunflower.*—Mr. Martin, in a paper by him to the Societe Therapeutique de France, affirms that the common sunflower, extensively cultivated, has the effect of neutralizing the unwholesome vapors which are so fatal to health and life in marshy districts. The Dutch, who live only by dyking and draining their low lands, and are therefore good authority, pronounce sunflower culture a specific for intermittent fever,—the scourge of Holland. They assert that it has disappeared from every district when the experiment has been tried. It is not yet known whether this is the result of its rapid growth producing oxygen, or whether it emits ozone and destroys those germs, animal and vegetable, which produce that miasma which brings fever in its train.—*The Pharmacist.*

---

*Test for the Purity of Chloral.*—When a fragment of pure hydrate of chloral is placed in a test tube or wine glass with a little water, and a few drops of liquor potassæ allowed to fall upon it, no discoloration takes place, and there is evolution of pure chloform, which can be detected by the odor. If the specimen experimented with is *impure*, a *dark* or *brown* reaction will result, and the odor evolved will be unpleasant. In this way a certain class of common impurities may be detected. In an article upon this important agent, to be published soon, we shall give a more extended account of it in all its relations.—*Boston Journal of Chemistry.*

*Undeveloped Eye Tooth.*—The second specimen exhibited by Dr. Sayre was a portion of an upper jaw containing an undeveloped eye-tooth. The specimen had the following history: Nine years ago a woman had presented herself with supposed cancer of the upper jaw. There was a tense shining swelling and apparent epulis of the upper jaw-bone. It was thought advisable to make exsection of the jaw. The tumor broke and blood escaped. There was considerable sloughing and more or less pain. The patient was discharged. A week ago she presented herself with a portion of the jaw still left. This was removed with a pair of forceps, and with it a large eye-tooth which had not been developed, and which was probably the source of all the irritation.—*Medical Record.*

---

*Osborn's Neuralgic Pill.*—T. C. Osborn, M. D., Greensboro. Alabama, writes to the editors of the *New Orleans Journal of Medicine*, that the subjoined combination is very effectual in cases of neuralgia: ℞. Zinci cyanuretum, gr. vj.; quiniæ sulphas, gr. ix.; morphiæ sulphas, gr. iss.; ext. belladonnæ, gr. iij. Ft. pilulæ No. vj. S. One pill every six hours until the pain is relieved.—*Drug. Cir. and Chem. Gaz.*

---

